# Selenium inhibits growth of trastuzumab-resistant human breast cancer cells via downregulation of Akt and beclin-1

**DOI:** 10.1371/journal.pone.0257298

**Published:** 2021-09-15

**Authors:** Joohyun Woo, Jong Bin Kim, Taeeun Cho, Eun Hye Yoo, Byung-In Moon, Hyungju Kwon, Woosung Lim

**Affiliations:** 1 Department of Surgery, Ewha Womans University College of Medicine, Seoul, South Korea; 2 Ewha Institute of Convergence Medicine, Seoul, South Korea; Columbia University, UNITED STATES

## Abstract

The response rate to treatment with trastuzumab (Tz), a recombinant humanized anti-HER2 monoclonal antibody, is only 12–34% despite demonstrated effectiveness on improving the survival of patients with HER2-positive breast cancers. Selenium has an antitumor effect against cancer cells and can play a cytoprotective role on normal cells. This study investigated the effect of selenium on HER2-positive breast cancer cells and the mechanism in relation to the response of the cells to Tz. HER2-positive breast cancer cell lines, SK-BR-3 as trastuzumab-sensitive cells, and JIMT-1 as Tz-resistant cells were treated with Tz and sodium selenite (selenite). Cell survival rates and expression of Her2, Akt, and autophagy-related proteins, including LC3B and beclin 1, in both cell lines 72 h after treatment were evaluated. Significant cell death was induced at different concentrations of selenite in both cell lines. A combined effect of selenite and Tz at 72 h was similar to or significantly greater than each drug alone. The expression of phosphorylated Akt (p-Akt) was decreased in JIMT-1 after combination treatment compared to that after only Tz treatment, while p-Akt expression was increased in SK-BR-3. The expression of beclin1 increased particularly in JIMT-1 after only Tz treatment and was downregulated by combination treatment. These results showed that combination of Tz and selenite had an antitumor effect in Tz-resistant breast cancer cells through downregulation of phosphorylated Akt and beclin1-related autophagy. Selenite might be a potent drug to treat Tz-resistant breast cancer by several mechanisms.

## Introduction

The human epidermal growth factor receptor 2 (HER2) gene, which encodes expression of transmembrane glycoprotein receptors with intracellular tyrosine kinase activity, is amplified in 25–30% of invasive breast cancers [1]. In normal cells, HER2 plays a role in the growth and division of cells, apoptosis, angiogenesis, and cell differentiation. However, overexpression of HER2 accelerates cell growth and promotes tumor development in several cancers. Therefore, clinically, HER2-positive breast cancers show a higher relapse rate, a higher metastatic potential, and a lower overall survival than HER2-negative breast cancers [2].

The prognosis of HER2-positive breast cancer has improved remarkably since trastuzumab (Tz), a recombinant humanized anti-HER2 monoclonal antibody, was developed. Treatment with Tz yields outcomes for HER2-positive early breast cancers similar to those of hormone receptor-positive breast cancers. Tz is known to have a cytotoxic effect through activation of antibody-dependent cellular cytotoxicity [2]. Furthermore, the therapeutic inhibits the shedding of the extracellular domain of HER2 that is proteolytically cleaved by metalloproteases when overexpressed [3]. HER2 downregulation is, therefore, induced and PI3K signaling pathway is negatively regulated. Tz can also inhibit tumor angiogenesis [2, 4].

The overall response rate of HER2-positive breast cancers to Tz is reported to be 12–34% for a median duration of 9 months; however, Tz is combined with other chemotherapeutic drugs, such as taxane for recurred or metastatic breast cancers, to increase cytotoxic efficacy. Adverse effects of chemotherapeutic agents provide limitations for the continuous use of the drugs in spite of a longer time to progression and better survival rate [5]. Several mechanisms explain the ways cancer cells obtain resistance to Tz. Loss of phosphatase and tensin homolog (PTEN) activity results in upregulation of AKT and Tz efficacy can decrease [4]. Likewise, alternative cell signaling mediated by insulin-like growth factor (IGF)- or by epidermal growth factor receptor (EGFR)-family pathways associated with transforming growth factor-α (TGF-α) can reduce HER2 inhibition [6].

According to recent studies, selenium is an essential micronutrient that has an antitumor effect in various cancers [7]. Selenium can induce mitochondria- and caspase-dependent apoptosis, cell cycle arrest, and DNA segmentation. Selenium can also selectively protect normal cells from cellular stress even though the chemopreventive effect of selenium including protection against oxidative damage is related to toxicity of selenium [8]. Some studies suggest that the action of selenium is different between transformed cells and normal cells [9]. Therefore, through combination with appropriate concentration of selenium considering it might cause toxicity toward normal cells, a higher dose regimen of chemotherapeutic agents can be provided [10].

Autophagy is an intracellular process whereby a portion of the cytoplasm and organelles are sequestered into a double membraned vesicle and fused with lysosomes for degradation of the enclosed materials. The latter pathway is crucial for cell survival and enables tumor cells to overcome stressors, the tumor microenvironment, and injuries caused by treatments such as endocrine therapy, chemotherapy, and radiation therapy [11].

Phosphatidylinositol-3 kinase (PI3K) signaling regulates cell growth, motility, and survival and is known to be deregulated in many cancers. Activation of this pathway can inhibit apoptosis and autophagy, mechanisms of programmed cell death, and can contribute to transformation of normal cells to tumor cells and, consequently, tumor growth [12]. PI3K signaling is also associated with resistance or chemosensitivity to a drug [13]. The serine/threonine kinase Akt is a major mediator of survival signals and has an ability to phosphorylate and inactivate downstream targets. Therefore, the PI3K/Akt signaling pathway is considered to be an important target for anticancer therapies.

Several studies have shown that selenium modulated Akt phosphorylation in several cancers and that inhibition of PI3K/Akt survival signaling is targeted by Tz’s antitumor activities [14–16]. Additionally, there are few published studies on the selenium action associated with regulation of Akt in breast cancer cells [16]. Therefore, this study aims to study the effect of selenium on treatment of HER2-positive breast cancer with Tz. Selenium can also change the autophagic activity regulated by mechanistic target of rapamycin (mTOR), a key molecule downstream of the PI3K/Akt signaling pathway [17]. We investigated the effect of selenium when combined with Tz in Tz-sensitive and–resistant cell lines. The mechanism by which selenium impacts the cells is focused on regulation of Akt and autophagic activity relative to apoptotic cell death.

## Materials and methods

This study was approved by the institutional review board of the Ewha Clinical Trial Center at Ewha University Medical Center (No. 2015-12-003-003). In this study, cell lines from human were used and the data was analyzed anonymously.

### Cell culture

MCF-10A cell line was purchased from the American Tissue Culture Collection (ATCC). These cells are derived from a 36 years old female with fibrocystic disease of mammary gland [18]. The cells were grown in DMEM/F12 with 5% FBS, 0.5 μg/mL hydrocortisone, 10 μg/mL insulin, 20 ng/mL EGF, 0.1 μg/mL cholera toxin, 2mM L-glutamine, 50 μg/mL gentamycin. MCF-7, estrogen receptor (ER)-positive and HER2-negative breast cancer cell line, was obtained from Korean Cell Line Bank (Seoul, Korea). These cells are derived from pleural effusion of a 51 years old Caucasian woman with breast cancer [19]. This cell line was maintained in growth media RPMI (Welgene, Korea) with 10% FBS (Welgen Korea) and 1% penicillin-streptomycin solution (Welgen, Korea).

SK-BR-3, HER2-positive and trastuzumab-sensitive breast cancer cell line, was obtained from Korean Cell Line Bank (Seoul, Korea). These cells are derived from pleural effusion of 43 years old Caucasian women with breast cancer [20]. JIMT-1, HER2-positive and trastuzumab-resistant breast cancer cell line, was purchased from AddexBio (San Diego, CA, USA). These cells are derived from pleural effusion of a 62 years old woman with breast cancer [21]. Cells were cultured in Dulbecco’s modified Eagle’s medium (DMEM; Gibco; Thermo Fisher Scientific, Inc., Waltham, MA, USA), supplemented with 10% fetal bovine serum (FBS; Gibco; Thermo Fisher Scientific, Inc.). Cultures were prepared by seeding 5 × 10^5^ cells into a Falcon^™^ Standard Tissue Culture Dish (Thermo Fisher Scientific, Inc.). Cells were incubated at 37°C in a humidified 5% CO2 atmosphere.

### Cell viability after drug treatment

Following culture of 5 × 10^5^ cells seeded in DMEM supplemented with 10% FBS for 24 h, cells were washed twice with PBS, and fresh medium was added. Tz (Herceptin^®^) was a gift of Roche Pharmaceuticals (Seoul, Korea) and sodium selenite (selenite) as a selenium compound was obtained from Sigma-Aldrich (St. Louis, MO, USA). Her2-positive cells, SK-BR-3 and JIMT-1 were treated with distilled water (control); Tz at 10 ng/mL, 100 ng/mL, 500 ng/mL, 1 μg/mL, 10 μg/mL and 100 μg/mL; selenite at 1 μM, 3 μM, 5 μM, and 10 μM; or Tz (1 μg/mL) plus selenite (1 μM, 3 μM, 5 μM, and 10 μM) for 72 h. As a control, non-malignant breast epithelial cells, MCF-10A and HER2-negative cells, MCF-7 were treated with distilled water (control); Tz at 1 μg/mL, 10 μg/mL; selenite at 5 μM, and 10 μM; or Tz (1 μg/mL) plus selenite (5 μM, and 10 μM) for 72 h. Viable cells were counted in a Neubauer chamber. Relative survival rates are shown as percentages of untreated control cells.

### Flow cytometry for cell death analysis

For cell death analysis, cells treated with distilled water (control), 1 μg/mL Tz, selenite (Sigma-Aldrich; Merck KGaA) (5 and 10 μM), or 1 μg/mL Tz plus selenium (5 μM, and 10 μM) for 72 h were collected by centrifugation at 300 x g for 3 min at 25°C. Annexin V staining was performed according to the manufacturer’s protocol (BD Biosciences, San Jose, CA, USA). After centrifugation, cells were washed with PBS and resuspended in binding buffer at 1 × 10^6^ cells/mL. An aliquot (100 μL) of the solution containing 1 × 10^5^ cells was transferred to a microcentrifuge tube, and 5 μL each of Annexin V-FITC and 1 μg/mL propidium iodide were added. After vortexing, cells were incubated for 1 h at 4°C in the dark, and 400 μL of binding buffer was added to each microcentrifuge tube. Flow cytometry was performed on a FACSCalibur system (BD Biosciences, San Jose, CA, USA) within 1 h.

### Expression pattern by immunoblotting

Following culture of 5 × 10^5^ cells seeded in DMEM supplemented with 10% FBS for 24 h, cells were washed twice with PBS and fresh medium was added. Cells were treated with distilled water (control), 1 μg Tz, selenite (Sigma-Aldrich; Merck KGaA) (5 and 10 μM), or 1 μg/mL Tz plus selenite (5 μM, and 10 μM) for 72 h. Total cell lysates were prepared in 200 μL of RIPA buffer according to the manufacturer’s protocol (Cell Signaling Technology, Beverly, MA). Protein concentrations were measured using the Bradford assay with the Bio-Rad Protein Assay kit (Bio-Rad Laboratories, Hercules, CA) according to the manufacturer’s directions. Equal amounts of proteins were separated by 10% sodium dodecyl sulfate-polyacrylamide gel electrophoresis (SDS-PAGE) and electrotransferred to Hybond-ECL nitrocellulose membrane (Amersham Bioscience, Buckinghamshire, UK). Blots of proteins labeled with anti-HER2 rabbit polyclonal antibodies (1:1000, Cat No: 2165S, Cell Signaling), anti-phospho-HER2 rabbit polyclonal antibodies (1:1000, Cat No: 2147S, Cell Signaling), anti-Akt rabbit polyclonal antibodies (1:1000, Cat No:SC8312, Santa Cruz Biotechnology), anti-phospho-Akt rabbit polyclonal antibodies (1:1000, Cat No:SC7985R, Santa Cruz Biotechnology), anti-LC3B rabbit polyclonal antibodies (1:1000, Cat No: 3868S, Cell Signaling), anti-beclin1 rabbit polyclonal antibodies (1:1000, Cat No: ab55878, Abcam), and anti-β-actin rabbit polyclonal antibodies (1:1000, Cat No:4970S, Cell Signaling) were washed with TBST buffer (Tris-buffered Saline, 0.2% Tween 20, Sigma, St. Louis, MO) and incubated for 1 h at room temperature with peroxidase-conjugated AffiniPure rabbit anti-mouse IgG (1:2500, Cat NO: 315-005-045, Jackson ImmunoResearch Laboratories, West Grove, PA) or peroxidase-conjugated AffiniPure mouse anti-rabbit IgG(1:2500, Cat NO: 211-005-109, Jackson ImmunoResearch Laboratories, West Grove, PA). Labeled proteins were detected using an enhanced chemiluminescence detection system (Amersham Bioscience).

### Statistics

Values are expressed as the mean ± standard error of at least triplicate independent experiments. Student’s t-test was used to compare between two groups and comparisons of more than two groups were analyzed by the one-way analysis of variance (ANOVA) test. Statistical analyses were performed using SPSS ver. 20.0 (SPSS Inc., Chicago, IL, USA). Two-sided p < 0.05(*) or p < 0.01(**) was considered to be statistically significant.

## Results

### Cytotoxic effects of Tz, selenite, and a combination of both on MCF-10A non-malignant cells and MCF-7 breast cancer cells

MCF-10A cells did not respond to Tz alone but decreased after treatment of selenite 5μM and 10μM with or without Tz. Effect of Se was dose-dependent (S1 Fig). MCF-7 cells also did not respond to Tz alone but decreased significantly at selenite 10μM and a combination of Tz 1μg/mL and selenite 10μM (S2 Fig). The effective dose of selenite in MCF-7, ER-positive and HER2-negative breast cancer cells was higher than in HER2-positive breast cancer cells.

### Cytotoxic effects of Tz, selenite, and a combination of both on SK-BR-3 and JIMT-1 breast cancer cells

**Fig 1:**
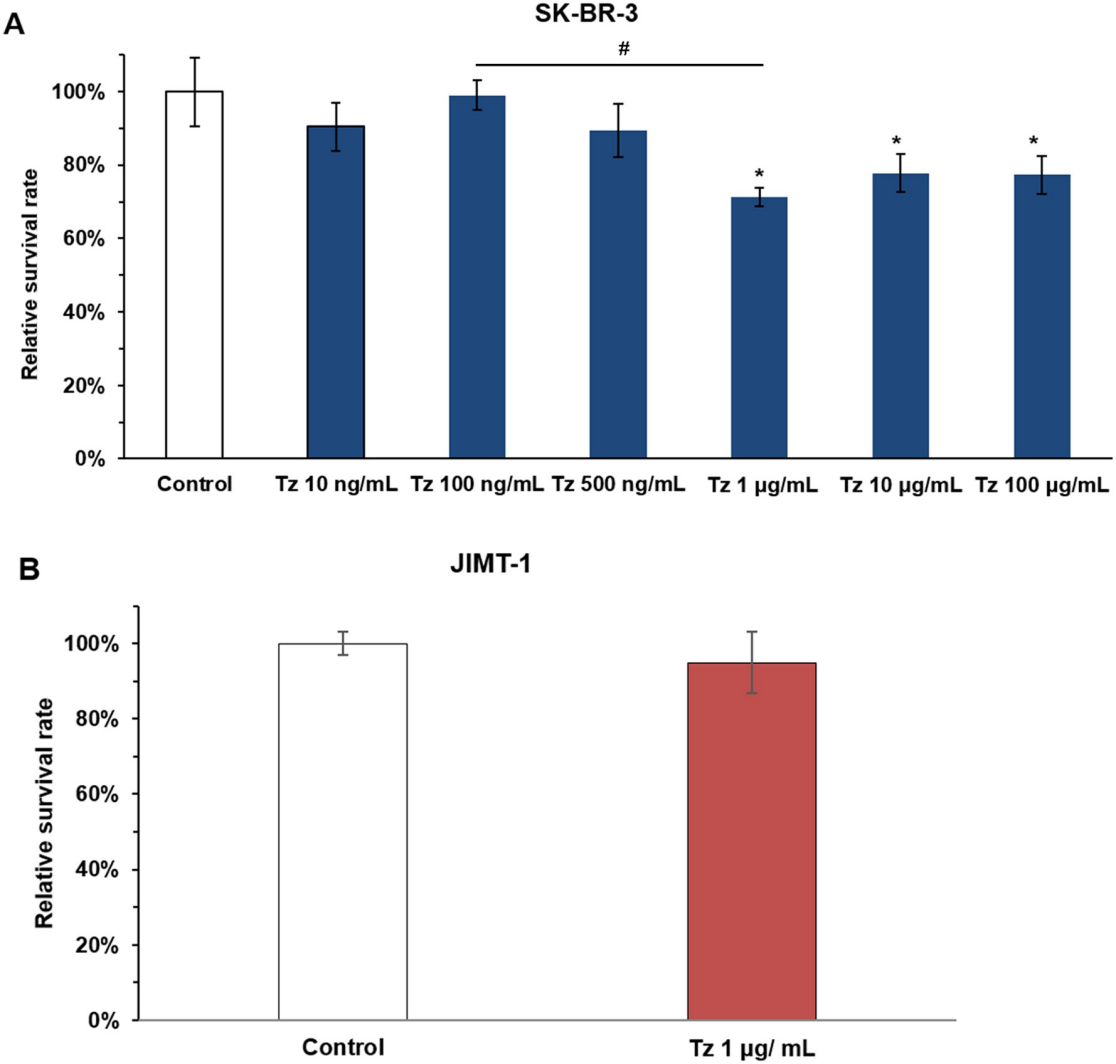
Cytotoxic effects of Tz on Tz-sensitive and -resistant breast cancer cells. (A) Response to Tz with dose increase in SK-BR-3 cell line. The optimal dose of Tz was established. (B) Resistance to Tz in JIMT-1 cell line. Cells were treated with distilled water (control) or Tz for 72 h. Viable cells were counted in a Neubauer chamber. Relative survival rates are shown as percentages of untreated control cells. Bars shows mean ± standard deviation of triplicates. One-way ANOVA followed by post Scheffe test was used for statistical analysis to compare between groups. (*P < 0.05 significantly different from control; ^#^ P < 0.05 significantly different from 1μg/mL Tz treatment) Student’s t-test was used for statistical analysis to compare Tz treatment with control in JIMT-1 cell line.

**Fig 2:**
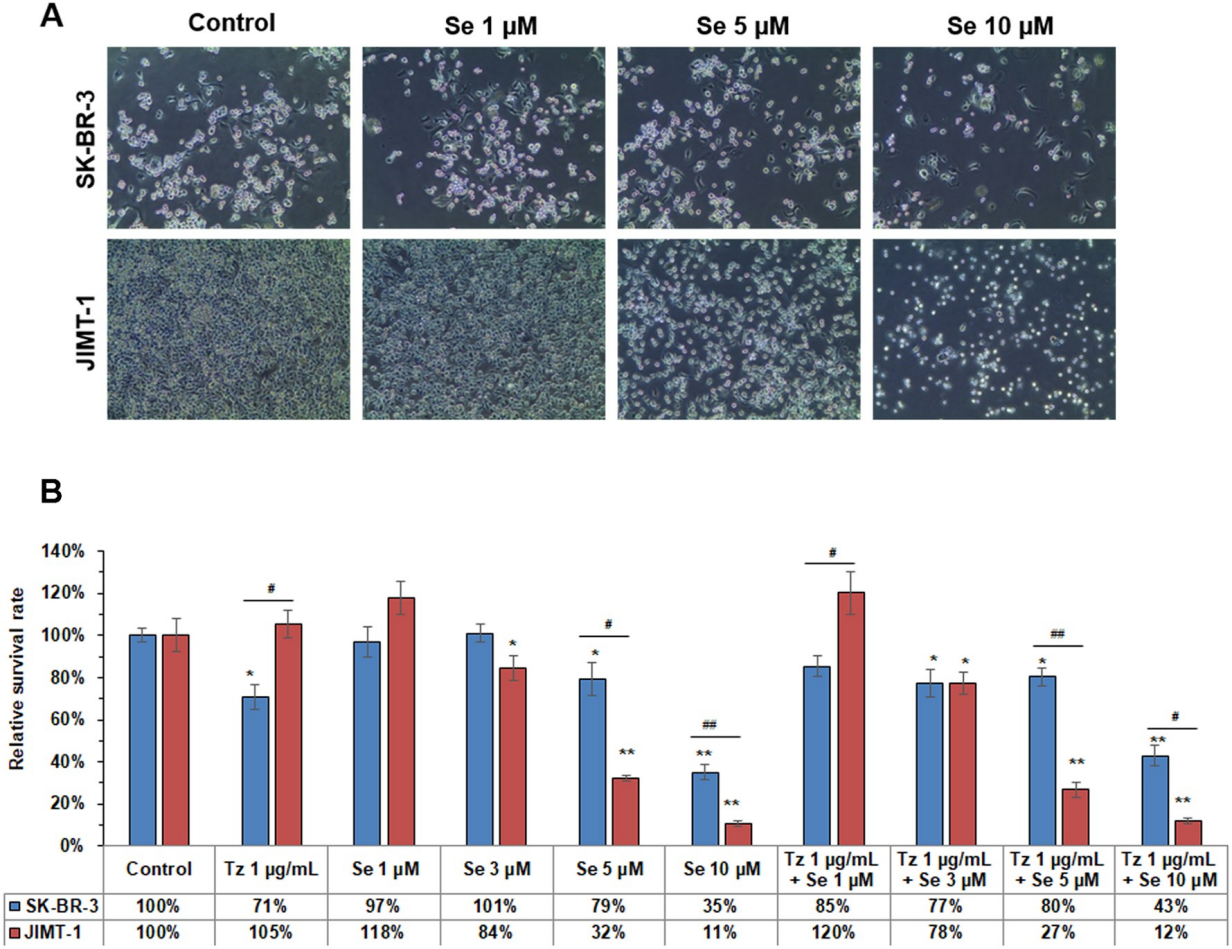
Cytotoxic effects of single and combination treatment of Tz and Se on Tz-sensitive and -resistant breast cancer cells. (A) Microscopic image after Se treatment in SK-BR-3 and JIMT-1 cells for 72 h. (B) Response to Se with dose increase in SK-BR-3 and JIMT-1 cell lines. Cells were treated with distilled water (control), Se at 1 μM, 3 μM, 5 μM and 10 μM, or Tz 1 μg/mL combined with Se at 1 μM, 3 μM, 5 μM or 10 μM for 72 h. Viable cells were counted in a Neubauer chamber. Relative survival rates are shown as percentages of untreated control cells. Bars shows mean ± standard deviation of triplicates. Control group received vehicle only. One-way ANOVA followed by post Scheffe test was used for statistical analysis to compare between groups. (*P < 0.05; **P < 0.01 significantly different from control in each cell line; ^#^ P < 0.05; ^##^ P < 0.01 significantly different between both cell lines).

The dose of Tz able to induce cell death in SK-BR-3 cells after 72 h of exposure was more than 1 μg/mL. Because cytotoxic activity was not dose dependent, 1 μg/mL Tz was chosen as a fixed dose when combined with selenite (Fig 1A). We confirmed that 1 μg/mL Tz did not show a cytotoxic effect on JIMT-1 cells after 72 h of incubation (Fig 1B). The concentration of selenite, which indicates cytotoxicity, was different in the two cells (Fig 2A). Compared with controls, 3 μM selenite decreased cell survival rate in JIMT-1 cells while 1 μM selenite did not show cytotoxic effect on both cells (Fig 2B). Treatment with 5 μM and 10 μM selenite for 72 h showed a concentration dependent cytotoxic effect on SK-BR-3 and JIMT-1 cells compared to the control group. In SK-BR-3 cells, the cell survival rate was further reduced by 10 μM selenite treatment relative to Tz treatment. In JIMT-1 cells, the cell survival rate was further decreased by 5 μM and 10 μM selenite treatment compared to Tz treatment. Moreover, 5 μM and 10 μM selenite were more cytotoxic toward JIMT-1 cells than SK-BR-3 cells (Fig 2).

Various concentrations of selenite after treatment with 1 μg/mL Tz for 72 h did not show an additive effect on SK-BR-3 cells (Fig 2B). On the contrary, combination treatment of Tz and 1 μM selenite decreased cytotoxicity in SK-BR-3 cells when compared to Tz alone without selenite. Cytotoxic effect of Tz combined with 5 μM or 10 μM selenite was less than that of 5 μM or 10 μM selenite alone. A lower cell survival rate was observed when a combination treatment of Tz and 10 μM selenite were used relative to Tz alone. In JIMT-1 cells, the combined effect of 3 μM, 5 μM, or 10 μM selenite with Tz for 72 h was significantly greater than Tz alone. The combined effect was slightly greater than selenite alone, although there was no statistical significance. Treatment with Tz combined with 5 μM and 10 μM selenite was more cytotoxic toward JIMT-1 cells than SK-BR-3 cells, similar to the results observed with selenite treatment (Fig 2B).

### Cytotoxicity via induction of apoptosis by selenite in JIMT-1

**Fig 3:**
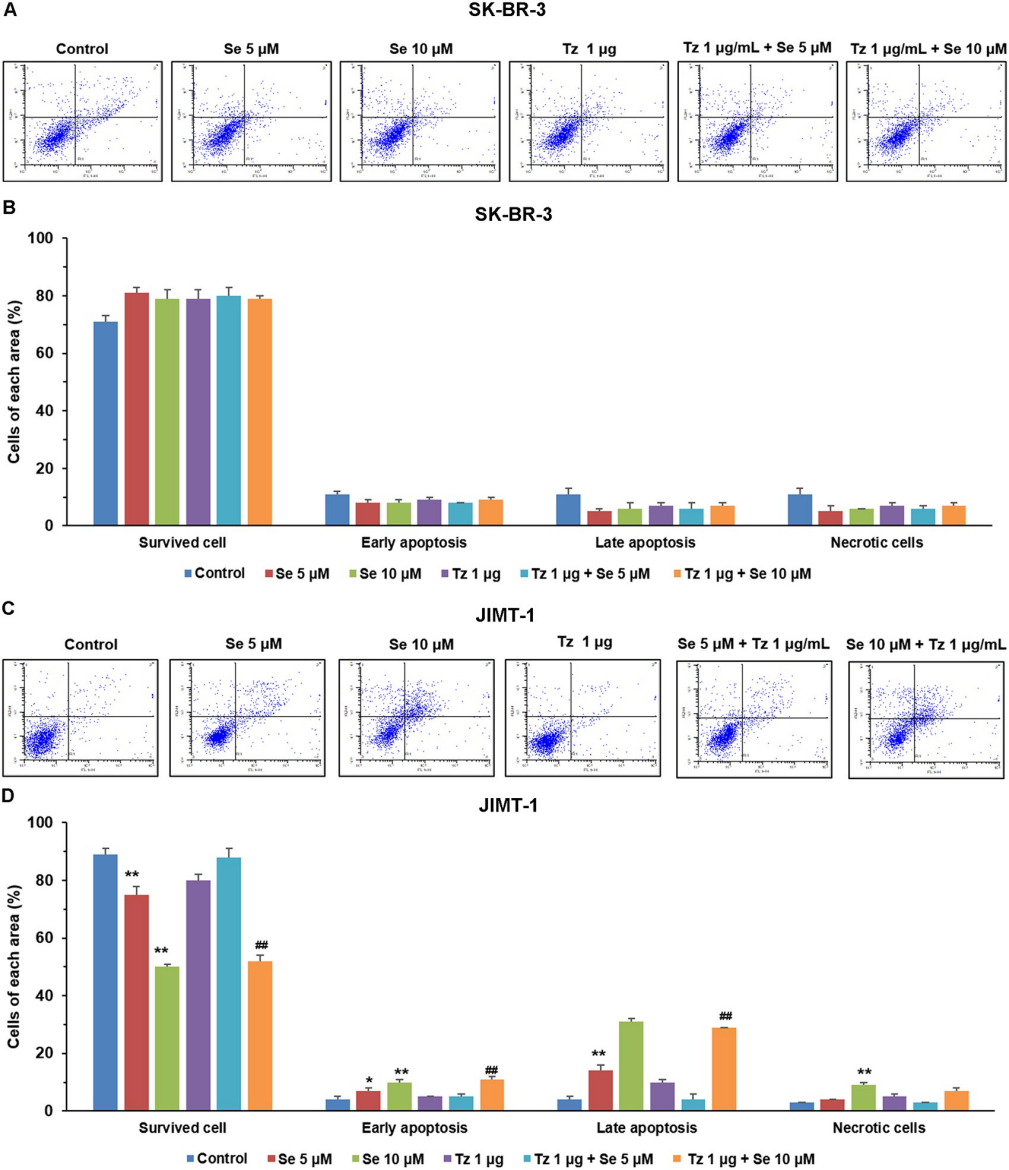
Effects on apoptosis in Tz-sensitive (A, B) and -resistant (C, D) breast cancer cell lines. Cells were treated with Se, Tz, or combinations of both drugs for 72 h. Cell deaths after treatments were analyzed by flow cytometry. Blue dots shows cell survival in the left lower quadrant, early apoptosis in right lower quadrant, late apoptosis in right upper quadrant and necrosis in left upper quadrant. Bars represent percentage of cells in each area analyzed by using flow cytometry. One-way ANOVA followed by post Scheffe test was used for statistical analysis to compare between groups. (*P < 0.05; **P < 0.01 significantly different from control; ^#^ P < 0.05; ^##^ P < 0.01 significantly different from Tz single treatment).

To investigate the mechanism of cell death including apoptosis and necrosis by selenite and the combination treatment, we performed cell death analysis after each treatment. There was no significant difference of apoptotic or necrotic cells in groups treated with 5 μM or 10 μM selenite alone when compared with control in SK-BR-3 cells. Induction of apoptosis or necrosis in groups treated with 1 μg/mL Tz alone or combination of 1 μg/mL Tz and selenite 5 μM or 10 μM was not observed in SK-BR-3 cells (Fig 3A and 3B). Meanwhile, groups treated with 5 μM or 10 μM selenite alone and combination of 1 μg/mL Tz and selenite 5 μM or 10 μM induced apoptosis in JIMT-1 cells when compared with control and Tz alone treatment (Fig 3C and 3D). There was no difference of necrotic cells between all treated groups and control in SK-BR-3 cells Selenite 10 μM induced necrosis when compared with control in JIMT-1 cells.

### Different regulation of Akt phosphorylation by selenite according to response to Tz

**Fig 4:**
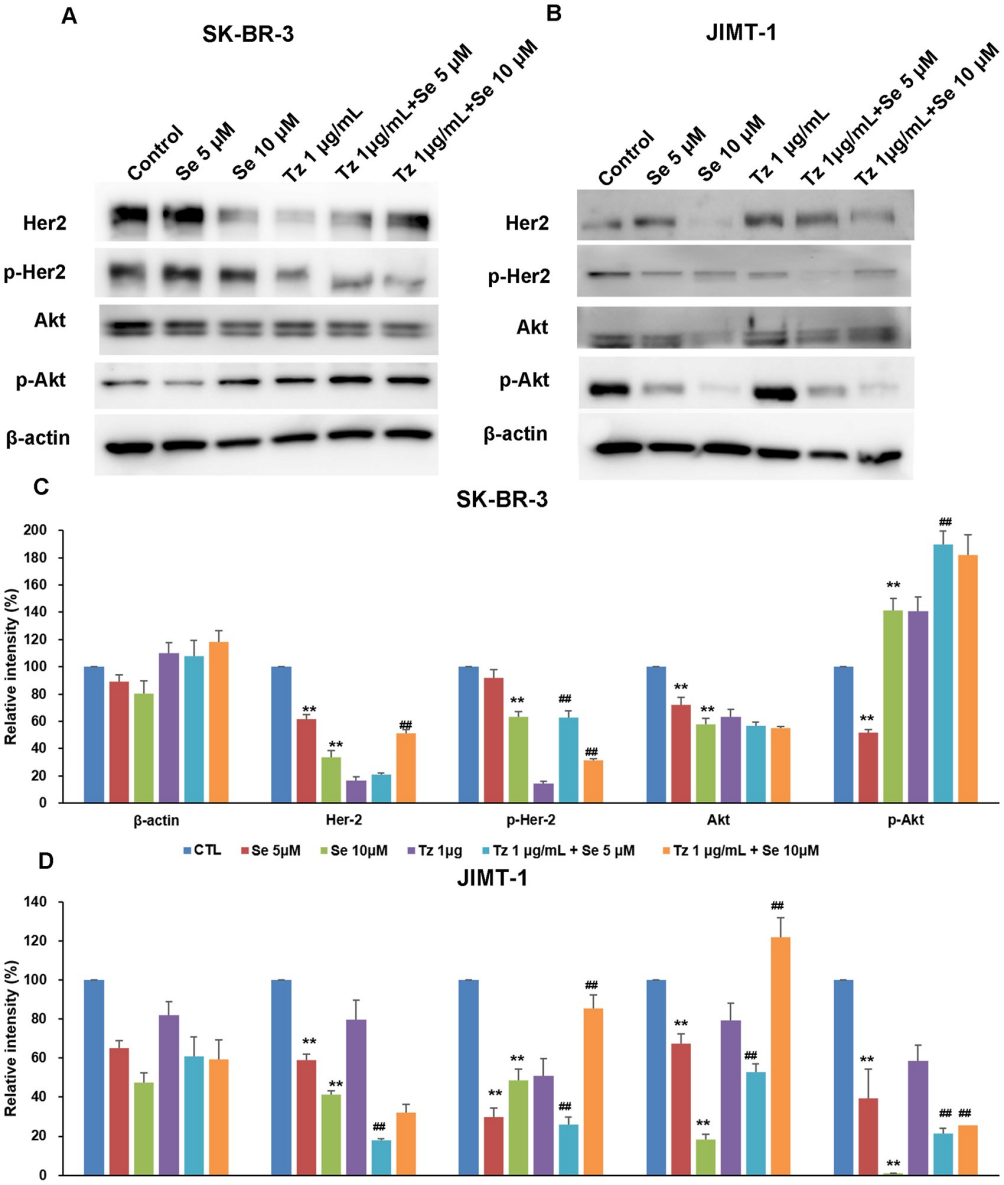
Changes in Her2 and Akt in Tz-sensitive (A, C) and -resistant (B, D) breast cancer cells. The data are presented as Her2, p-Her2, Akt, and p-Akt expressed relative to control after treatment of Se, Tz, and combinations of both drugs for 72 h by Western blot analysis and densitometry analysis. Similar results were observed in replicate experiments. Protein expression levels (relative to β-actin) were determined. One-way ANOVA followed by post Scheffe test was used for statistical analysis to compare between groups. (*P < 0.05; **P < 0.01 significantly different from control; ^#^ P < 0.05; ^##^ P < 0.01 significantly different from Tz single treatment). Effects of Se on Her-2 and Akt in SK-BR-3 and JIMT-1 were opposed to each other.

In both cell lines, expression of phosphorylated Her2 (p-Her2) decreased after treatment with Tz compared to the control group (Fig 4). In SK-BR-3 cells, selenium only treatment did not change expression of p-Her2 compared to the control group nor did treatment with selenium combined with Tz compared to Tz only. In contrast, in JIMT-1 cells, selenite only treatment decreased expression of p-Her2 compared to the control group as did treatment with selenite combined with Tz relative to Tz only.

Treatment with Tz for 72 h upregulated Akt phosphorylation in SK-BR-3 cells but downregulated Akt phosphorylation in JIMT-1 cells compared to the control group. In SK-BR-3 cells, treatment with selenite for 72 h slightly upregulated Akt phosphorylation compared to the control group but not as much as treatment with Tz. In JIMT-1, treatment of selenite markedly downregulated Akt phosphorylation as compared to treatment with Tz. The combination of selenite and Tz increased phosphorylated Akt (p-Akt) in SK-BR-3 cells but decreased p-Akt in JIMT-1 cells compared to treatment with Tz.

### Inhibition of beclin1-dependent autophagy by selenite in JIMT-1 cells

**Fig 5:**
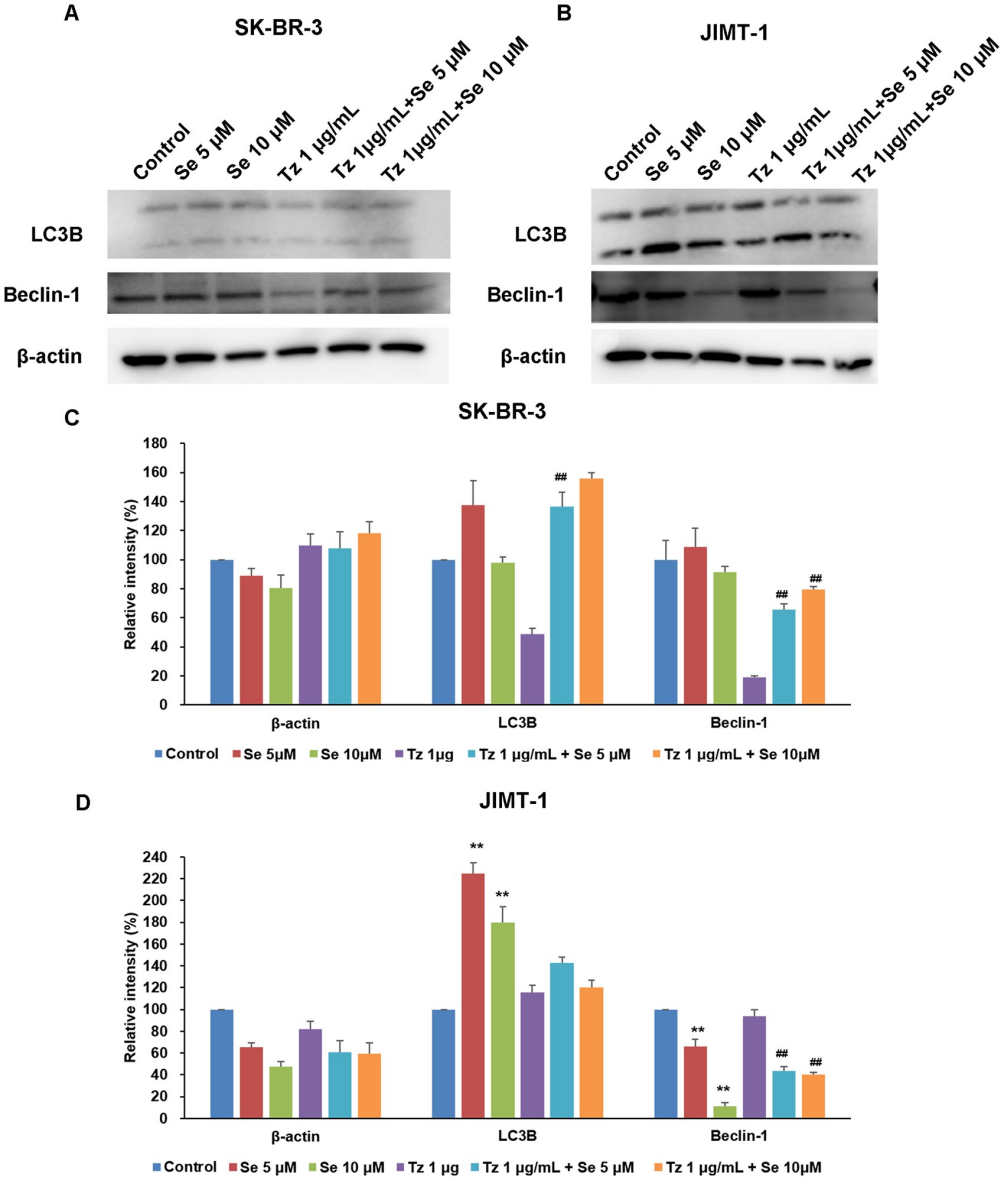
Changes of autophagic activity in Tz-sensitive (A, C) and -resistant (B, D) breast cancer cells. Autophagic activity was detected with Western blot analysis and densitometry analysis using LC3B and Beclin-1 antibodies in both cell lines after treatment of Se, Tz, and combinations of both drugs for 72 h. Similar results were observed in replicate experiments. Protein expression levels (relative to β-actin) were determined. One-way ANOVA followed by post Scheffe test was used for statistical analysis to compare between groups. (*P < 0.05; **P < 0.01 significantly different from control; ^#^ P < 0.05; ^##^ P < 0.01 significantly different from Tz single treatment). Inhibition of Beclin-1-related autophgic activity was shown in JIMT-1 cell line.

In both cell lines, treatment with selenite did not change beclin-1 expression compared to the control group (Fig 5). In JIMT-1 cells, Tz increased expression of beclin-1 compared to the control group. As compared to Tz treatment, beclin-1 was downregulated by treatment with selenite and Tz combined. In contrast, in SK-BR-3 cells, Tz decreased expression of beclin1, which increased after selenite was combined with Tz (Fig 5).

## Discussion

Upregulation of PI3K/Akt signaling has been suggested as a biomarker of resistance to Tz [22]. Thus, the PI3K/Akt pathway can be a potential target to overcome resistance to Tz. The present study revealed that selenium can downregulate p-Akt in Tz-resistant breast cancer cells. In other studies, selenium reversed an increased activity of Akt by doxorubicin and induced apoptosis of cancer cells in MCF-7 breast cancer cells [16]. Additionally, selenium deactivated Akt in prostate cancer cells via calcineurin [15]. In contrast, we observed that selenium induced Akt activity in Tz-sensitive breast cancer cells. This result could be associated with upregulation of p-Akt induced after treatment with Tz. In other words, the change of Akt activity induced by selenium was different between Tz-sensitive and Tz-resistant breast cancer cells. This finding may mean that the mechanism of action of selenium can differ depending on responsiveness to treatment and selenium can be used to overcome drug resistance. A recent study showed that selenium was unable to sensitize MCF-7 breast cancer cells to doxorubicin when Akt was activated. Upregulation of Akt phosphorylation by doxorubicin occurs concurrently with an increase of the Akt phosphorylated substrates, which lose pro-apoptotic capabilities [23]. In this study, a combination of selenium and Tz was used to increase the cytotoxic effect of Tz on breast cancer cells in which Akt had been downregulated.

The PI3k/Akt pathway is related to both apoptosis and autophagy. Akt acts as a direct molecule in the mechanism of tumor formation and regulates autophagy. Beclin-1, a mammalian orthologue of the yeast autophagy-related gene Atg6, induces autophagy to inhibit tumorigenesis and is associated with the development of several cancers [24]. Beclin1 seems to be a direct target of phosphorylation by Akt, and this phosphorylation functions in autophagy inhibition, oncogenesis, and the formation of an autophagy-inhibitory beclin-1/14-3-3/vimentin intermediate filament complex [25].

Along with apoptotic cell death, autophagic cell death is one of the programmed cell deaths. Because the two mechanisms are linked with each other, understanding changes of autophagic activity is important. Although evidence with regards to the induction of apoptosis by selenium has been accumulated, studies on the effect of selenium on autophagy has led to variable results. The relationship between apoptosis and autophagy is complicated and is not well understood [26]. The relationship appears to change according to the situation of the cell, including treatment by and response to drugs.

**Fig 6:**
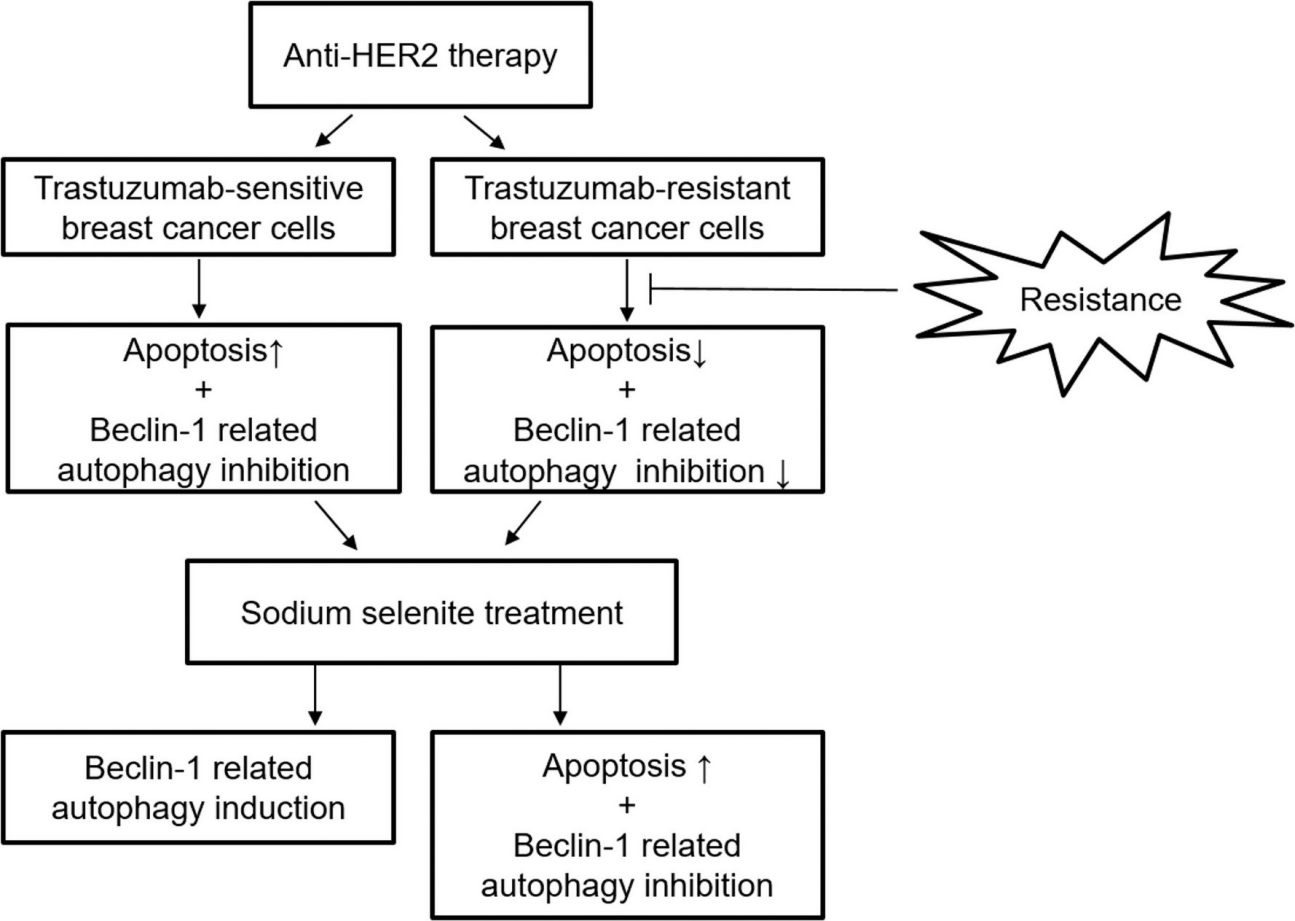
A schematic diagram of inhibitory effect of selenium on HER2-positive breast cancer cells.

In the present study, selenium enhanced the antitumor effect of Tz on Tz-resistant breast cancer cells via inhibition of beclin-1-related autophagic activity. Also, beclin-1-related autophagic activity by selenium was dependent on the response of cells to Tz (Fig 6). In NB4 cells, an acute promyeloyctic leukemia cell line, and in HCT116 cells, colorectal cancer cells, selenium inhibited autophagy and increased apoptosis via downregulation of p-Akt [10, 27]. This result is consistent with findings of the present study in Tz-resistant breast cancer cell lines. On the other hands, selenium induced autophagy in colorectal cancer cells in vitro and in vivo and inhibited apoptosis. However, further autophagy activation increased apoptosis [28]. The present study found that selenium induced beclin-1-related autophagy in Tz-sensitive breast cancer cells after treatment with selenium combined with Tz. The cytotoxic effect of selenium was less in Tz-sensitive breast cancer cells than in Tz-resistant breast cancer cells. This finding could result from antagonizing apoptotic cell death of autophagy induced by selenium in Tz-sensitive breast cancer cells.

The cytotoxicity of selenium is different between normal and cancer cells. Selenium inhibited the growth of human breast cancer cells, but the same or higher concentrations used with cancer cells did not affect non-cancerous cells. Also, parenteral administration of selenium reduced the rate of tumor growth without harmful effects on nude mice [7]. Moreover, because selenium can play a role in the protection of normal cells, as shown in vivo, selenium might be more helpful in the treatment of Tz-resistant cancers. Selenium inhibited the development of mammary tumors induced by a reliable tumor initiator in rat breast tissues through carcinogen-DNA adduct formation [29]. In normal cells, selenium can inactivate free radicals and protect against oxidative damage [30]. Meanwhile, selenium may cause toxicity in normal cells. There is little research evidence, even in vitro, for toxicity of selenium in normal cells. Published reports describe serum selenium of 400–30,000 μg/L is associated with serious side effect although there are some problems in measuring the concentration [31]. However, the degree of toxicity by selenium in normal cells can be different from that in cancer cells at the same concentration of selenium. The cancer-derived cells obtained from prostate cancer patients were significantly more sensitive to selenite-induced apoptosis than the corresponding normal cells [32]. Meanwhile, Zhang et al. showed cytotoxicity of sodium selenite on both cancerous and non-cancerous colon cells [33]. In our study, cytotoxic effect of sodium selenite was observed on non-tumorigenic epithelial cells. Therefore, determining the concentration of selenium that enhances cytotoxicity in cancer cells without toxic effect on normal cells would be important. Also, as different forms of selenium have much different magnitude of effect, further studies with other forms of selenium is needed.

Autophagic activity can vary according to response to treatment. Autophagy suppresses tumor development at the early stage and promotes development at the advanced stage. Furthermore, against anticancer treatment, autophagy functions as a protector of some cancer cells and induces cell death [24]. In the present study, beclin1-related autophagy was increased in Tz-resistant breast cancer cells after Tz treatment, which was different from Tz-sensitive breast cancer cells. Induction of autophagy can indicate resistance to antitumor treatment.

The anticancer effect of selenium in HER2-positive breast cancer cells was confirmed. It is consistent with the results from different subtype of breast cancer cells but the concentration of showing anticancer effect in MCF-7 cells, which are ER-positive and HER2-negative, was different from that in HER2-positive breast cancer cells. Vadgama et al. reported that SK-BR-3 cells derived from an aggressive tumor showed more significant inhibition compared to MCF-7 cells [34]. The lower inhibitory effect of selenium on MCF-7 cells was observed in our study. The effect of selenium used alone was greater in Tz-resistant breast cancer cells. Selenium combined with Tz was also more effective with Tz-resistant breast cancer cells than Tz-sensitive cancer cells. An in vitro study found that selenium attached to Tz can induce cell death in HER2-positive breast cancer with Tz resistance [35]. Enhancement or synergism of the antitumor effect on cancer cells has been reported for selenium combined with several chemotherapeutic drugs, such as doxorubicin, paclitaxel and docetaxel [36]. The increased cytotoxicity was shown to be a result of an induction of apoptosis and cell cycle arrest [10, 36]. We demonstrated that the cytotoxic effect and mechanism of selenium in HER2-positive breast cancer cells depends on responsiveness to Tz. Furthermore, we found that there was no statistically significant synergism of selenium and Tz, but that selenium had an additive inhibitory effect and did not antagonize the effect of Tz in HER2-positive breast cancer cells, regardless of the response to Tz.

We showed that selenium had an antitumor effect in Tz-resistant breast cancer cells through downregulation of Akt. Combination of Tz and selenium inhibited beclin-1-related autophagy in Tz-resistant breast cancer cells. Selenium might be a potent drug to treat Tz-resistant breast cancer. However, further investigation including in vivo studies are needed to identify other pathways linked to apoptosis and autophagy by selenium and to understand the mechanism at the molecular level. Also to identify whether downregulation of Akt induce the suppression of beclin-1-related autophagy using Akt inhibitor or autophagy inhibitor in Tz-sensitive and–resistant cells might help to clarify findings of this study.

## Supporting information

S1 Fig Cytotoxic effects of Tz, selenite, and a combination of both on MCF-10A non-malignant cells. (A) Microscopic image after each treatment in MCF-10A for 72 h. (B) Response to Se with dose increase in in MCF-10A cell lines. Cells were treated with distilled water (control), Se at 5 μM and 10 μM, Tz 1 μg/mL, Tz 10 μg/mL, or Tz 1 μg/mL combined with Se at 5 μM or 10 μM for 72 h. Viable cells were counted in a Neubauer chamber. Relative survival rates are shown as percentages of untreated control cells. Bars shows mean ± standard deviation of triplicates. Student’s t-test was used for statistical analysis to compare control and treated groups. Statistical significance is represented with asterisks (* P < 0.05, **P<0.01). Scale bar, 200μm.
(TIF)

S2 Fig Cytotoxic effects of Tz, selenite, and a combination of both on MCF-7 breast cancer cells. (A) Microscopic image after each treatment in MCF-7 for 72 h. (B) Response to Se with dose increase in in MCF-7 cell lines. Cells were treated with distilled water (control), Se at 5 μM and 10 μM, Tz 1 μg/mL, Tz 10 μg/mL, or Tz 1 μg/mL combined with Se at 5 μM or 10 μM for 72 h. Viable cells were counted in a Neubauer chamber. Relative survival rates are shown as percentages of untreated control cells. Bars shows mean ± standard deviation of triplicates. Student’s t-test was used for statistical analysis to compare control and treated groups. Statistical significance is represented with asterisks (* P < 0.05, **P<0.01). Scale bar, 200μm.
(TIF)

S1 Raw images Raw images of Western blot from Figs 4 and 5. (PDF)
