# Supplementary figures and images for: Selenium inhibits growth of trastuzumab-resistant human breast cancer cells via downregulation of Akt and beclin-1
Source: PLoS One. 2021 Sep 15;16(9):e0257298. doi: 10.1371/journal.pone.0257298 (PMC8443054; doi:10.1371/journal.pone.0257298)

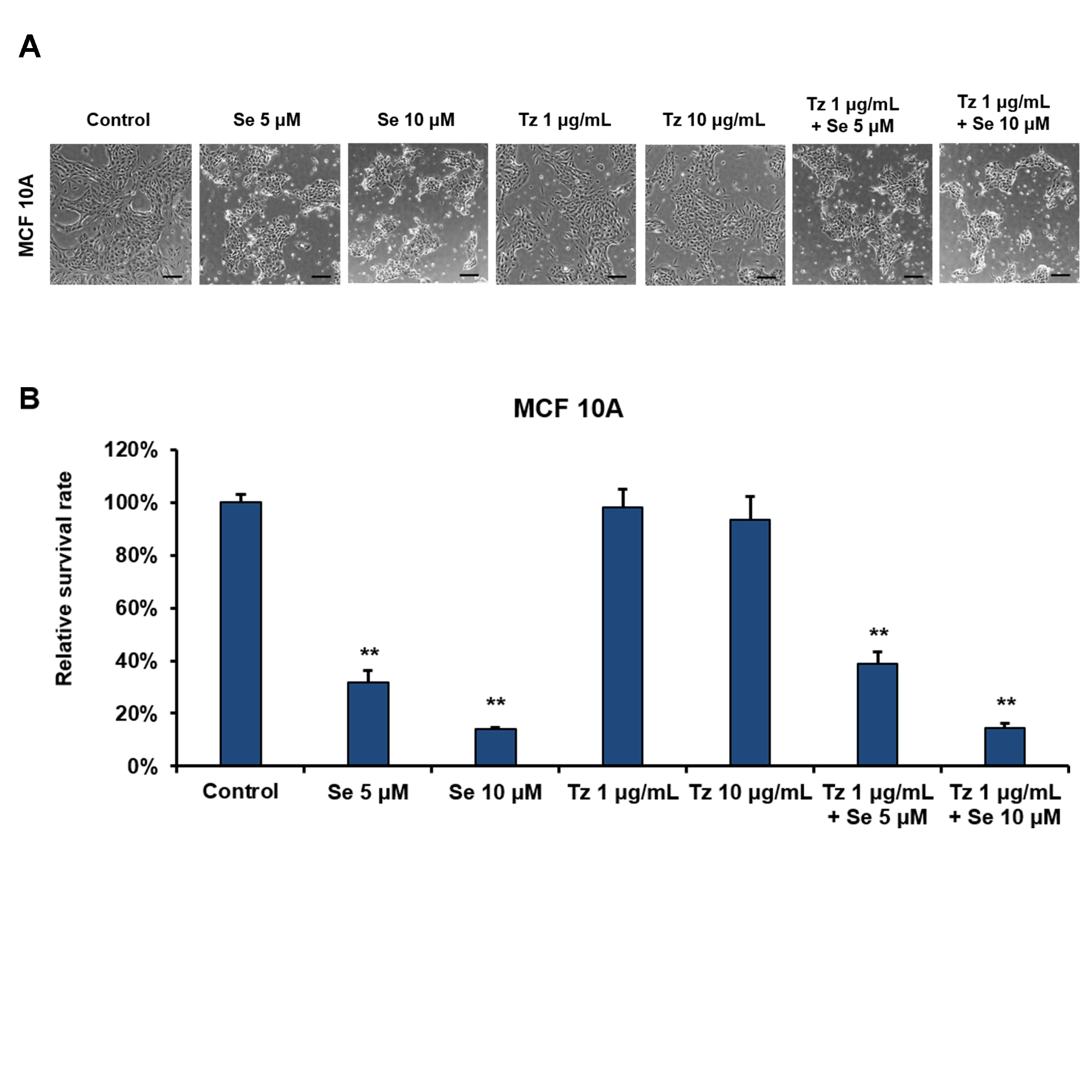

Supplement: S1 Fig — (A) Microscopic image after each treatment in MCF-10A for 72 h. (B) Response to Se with dose increase in in MCF-10A cell lines. Cells were treated with distilled water (control), Se at 5 μM and 10 μM, Tz 1 μg/mL, Tz 10 μg/mL, or Tz 1 μg/mL combined with Se at 5 μM or 10 μM for 72 h. Viable cells were counted in a Neubauer chamber. Relative survival rates are shown as percentages of untreated control cells. Bars shows mean ± standard deviation of triplicates. Student’s t-test was used for statistical analysis to compare control and treated groups. Statistical significance is represented with asterisks (* P < 0.05, **P<0.01). Scale bar, 200μm. (TIF) [file pone.0257298.s001.tif]

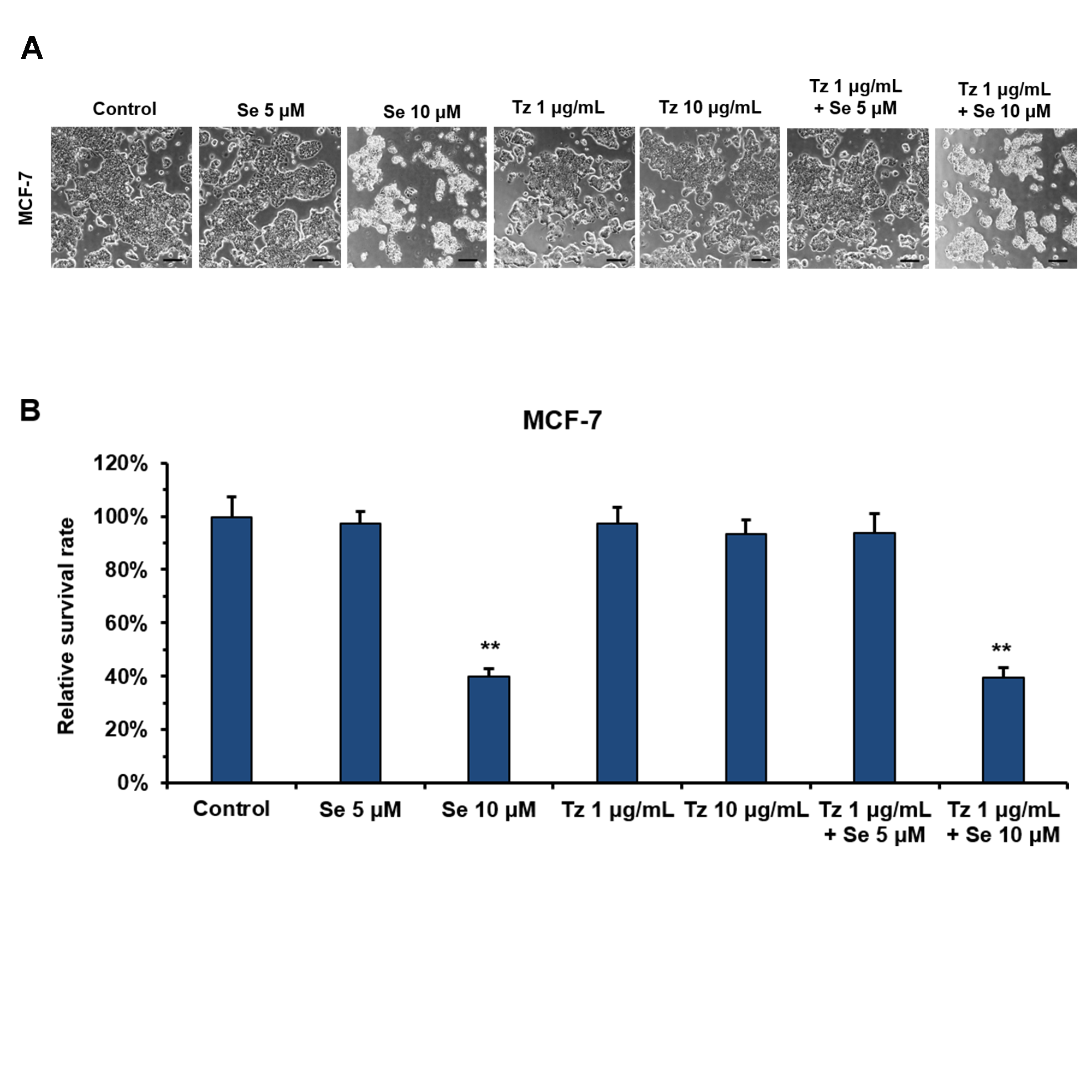

Supplement: S2 Fig — (A) Microscopic image after each treatment in MCF-7 for 72 h. (B) Response to Se with dose increase in in MCF-7 cell lines. Cells were treated with distilled water (control), Se at 5 μM and 10 μM, Tz 1 μg/mL, Tz 10 μg/mL, or Tz 1 μg/mL combined with Se at 5 μM or 10 μM for 72 h. Viable cells were counted in a Neubauer chamber. Relative survival rates are shown as percentages of untreated control cells. Bars shows mean ± standard deviation of triplicates. Student’s t-test was used for statistical analysis to compare control and treated groups. Statistical significance is represented with asterisks (* P < 0.05, **P<0.01). Scale bar, 200μm. (TIF) [file pone.0257298.s002.tif]
