# Supplementary material for: Selenium inhibits growth of trastuzumab-resistant human breast cancer cells via downregulation of Akt and beclin-1
Source: PLoS One. 2021 Sep 15;16(9):e0257298. doi: 10.1371/journal.pone.0257298 (PMC8443054; doi:10.1371/journal.pone.0257298)
Supplement: S1 Raw images — (PDF) [file pone.0257298.s003.pdf]

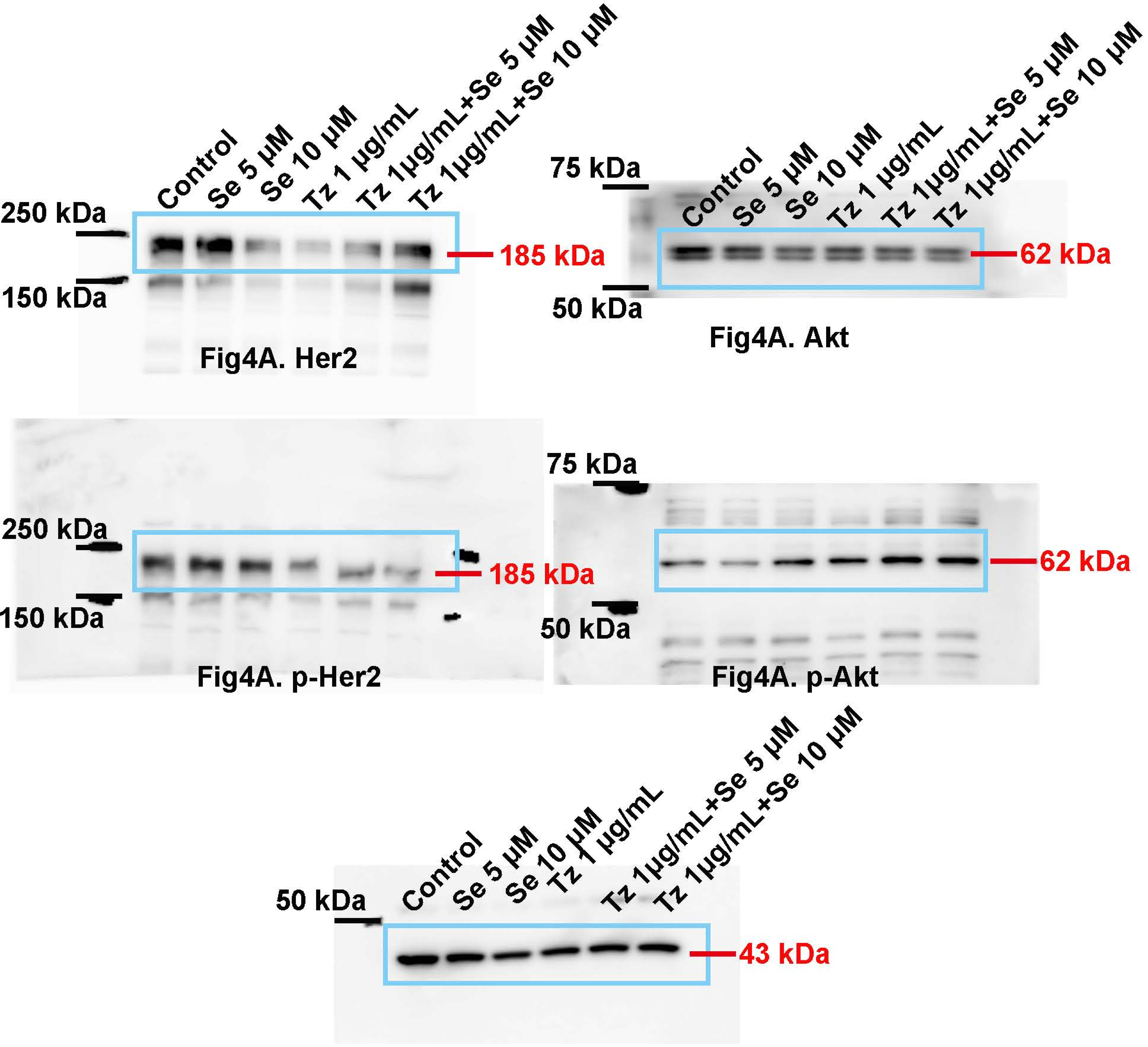

The images were scanned with ImageQuantTM LAS 4000 mini.

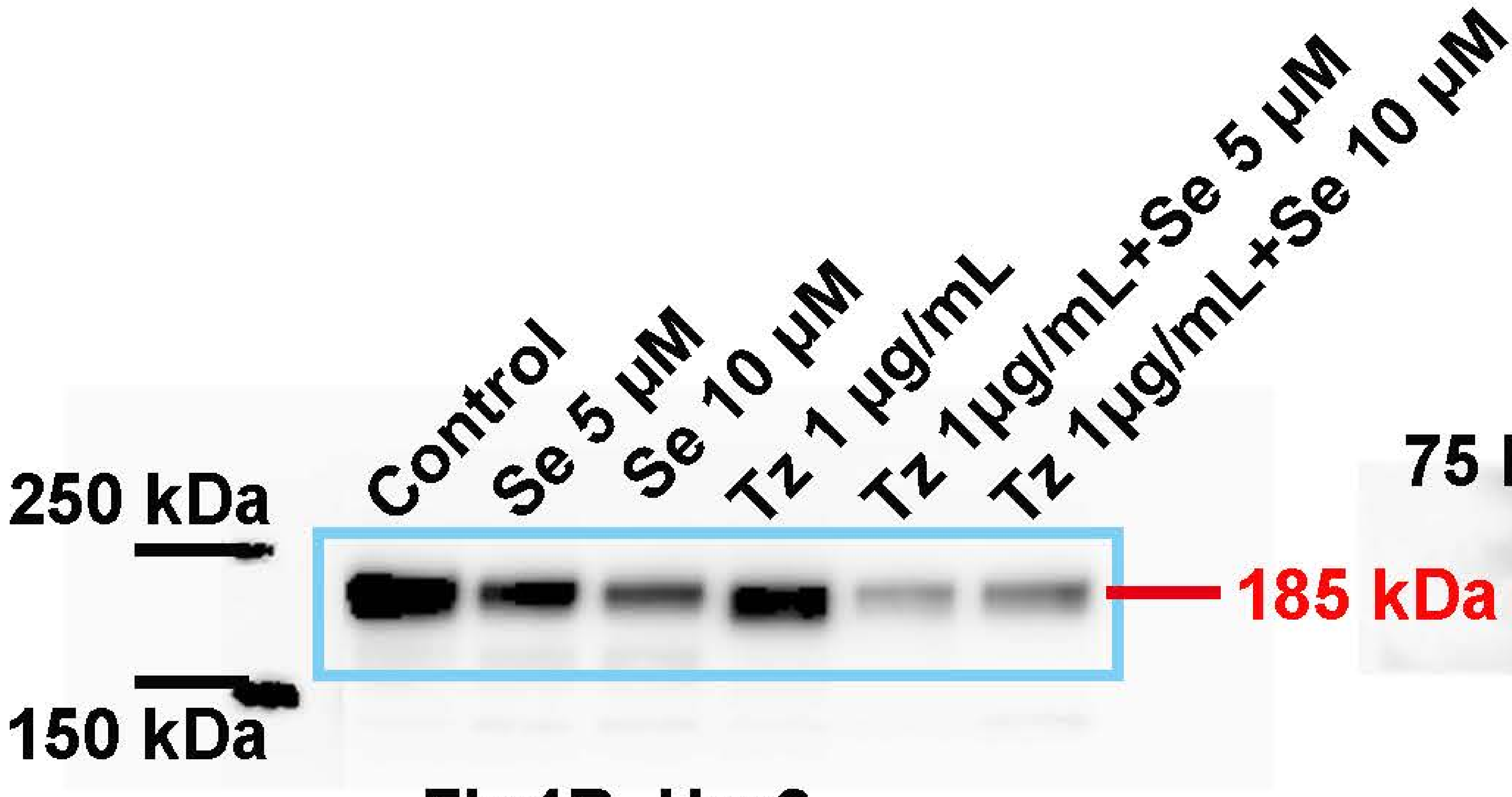

Fig4B. Her2

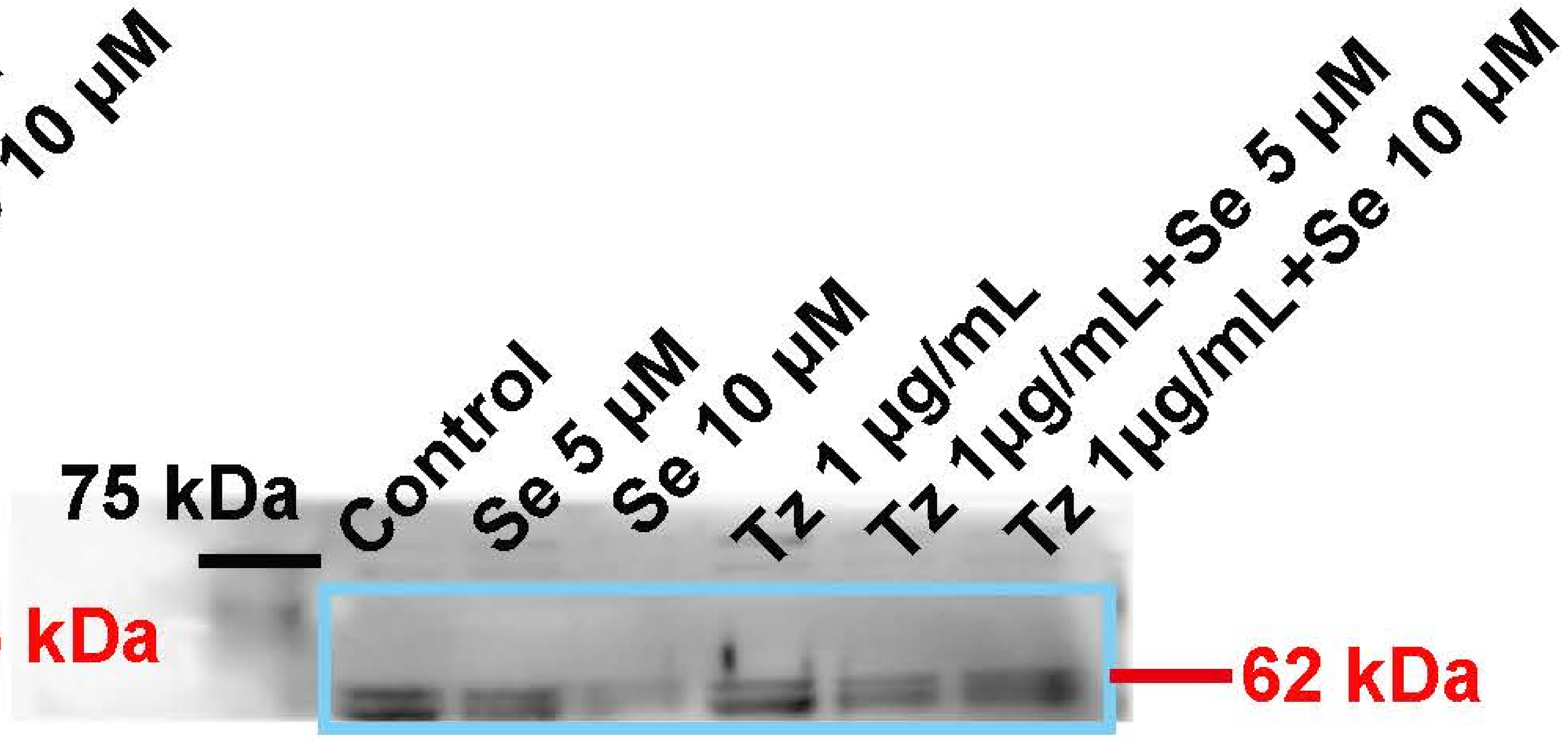

Fig4B. Akt

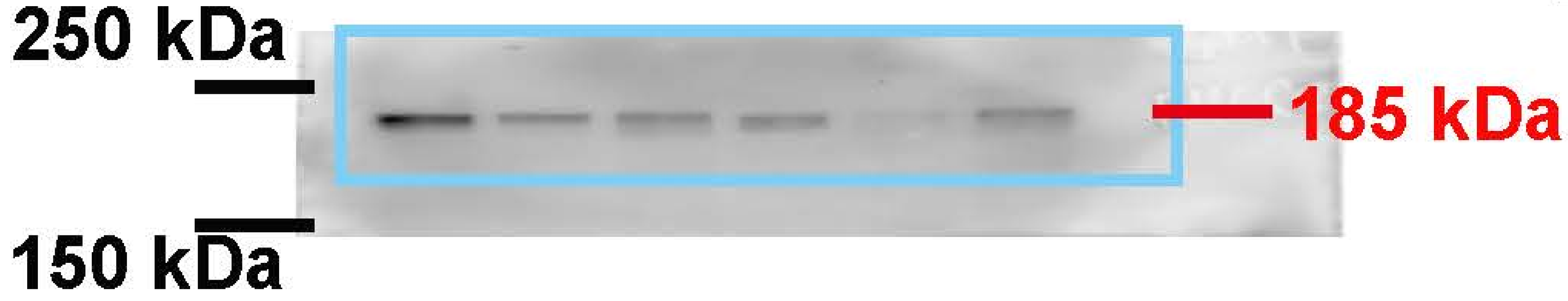

Fig4B. p-Her2

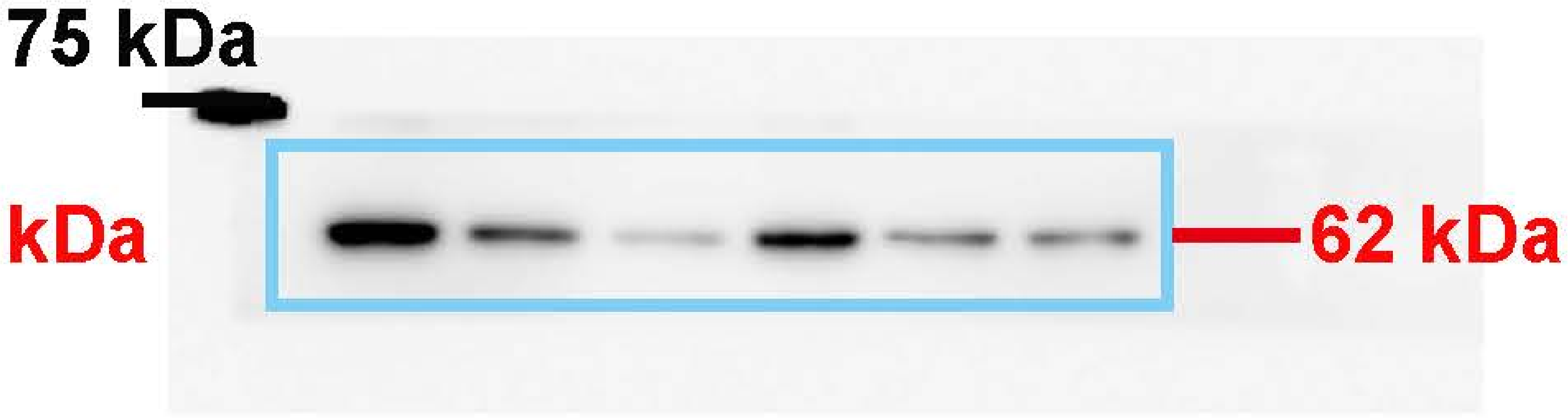

Fig4B. p-Akt

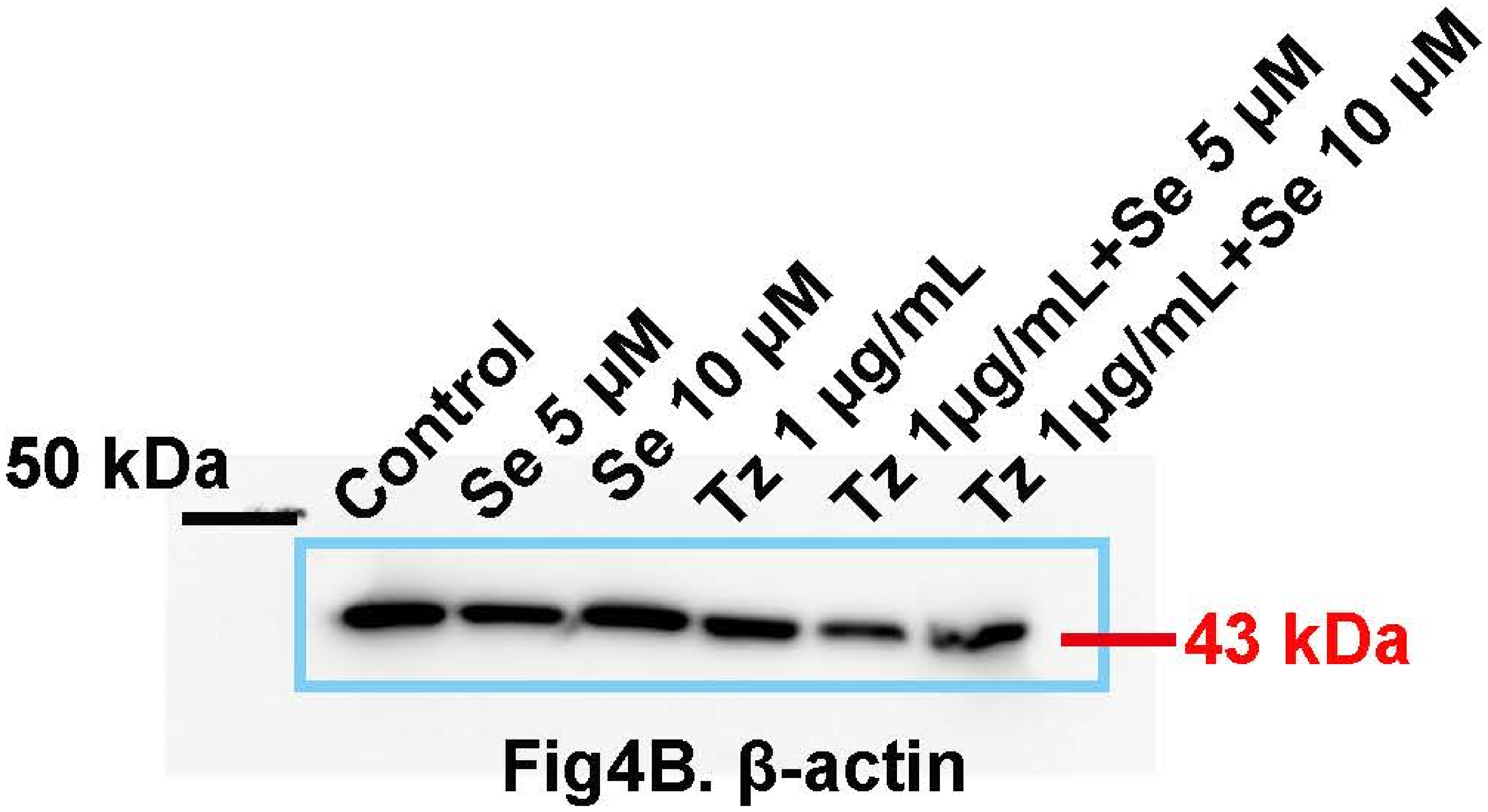

Fig4B. β-actin

The images were scanned with ImageQuantTM LAS 4000 mini.

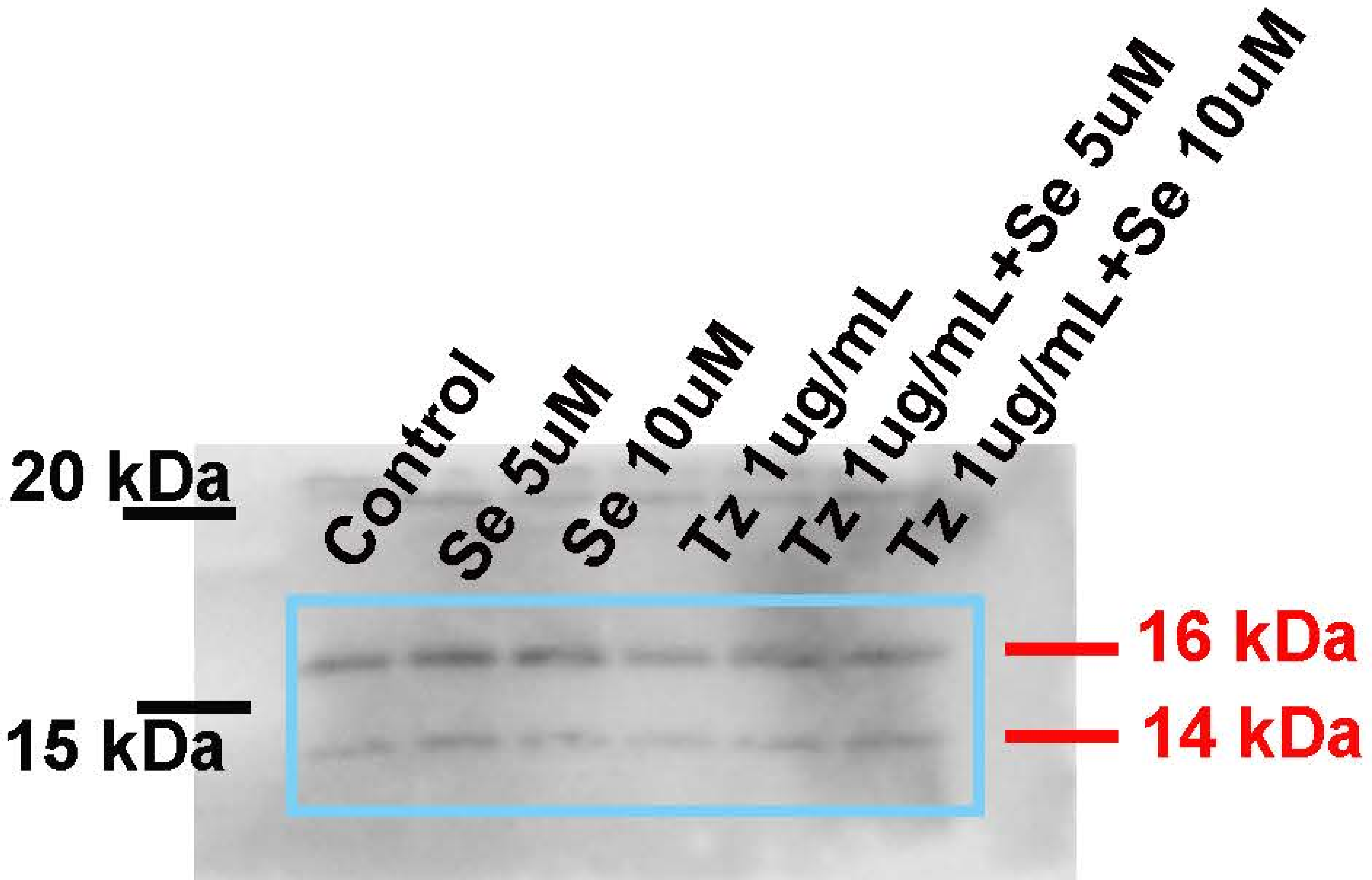

Fig 5A. LC3B

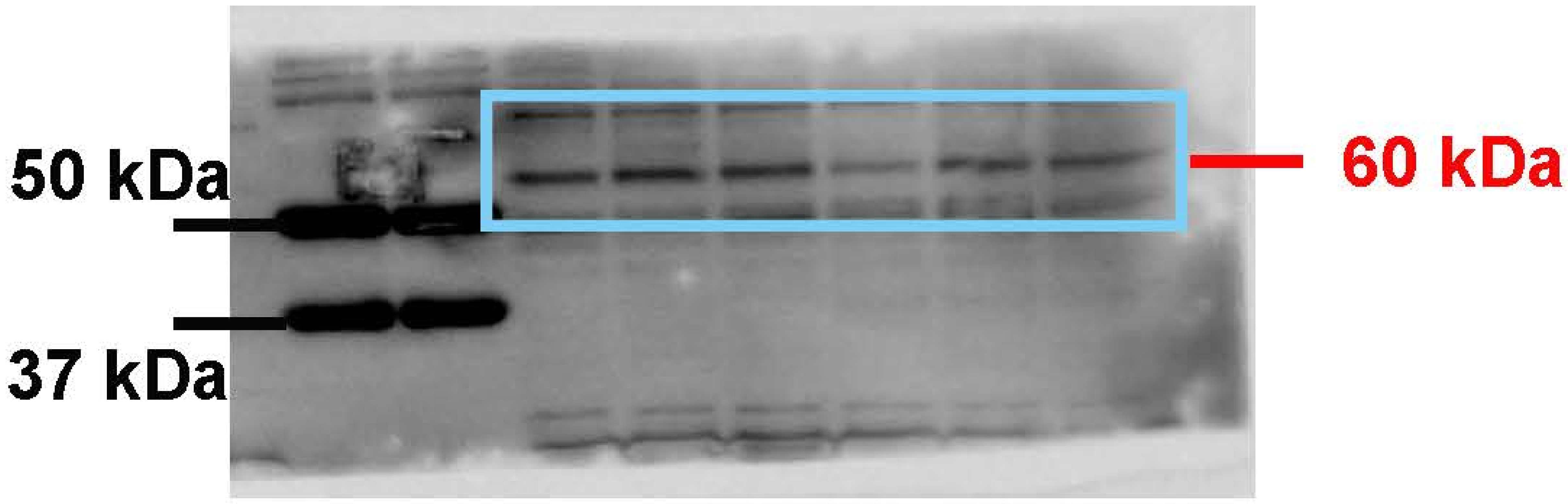

Fig 5A. Beclin-1

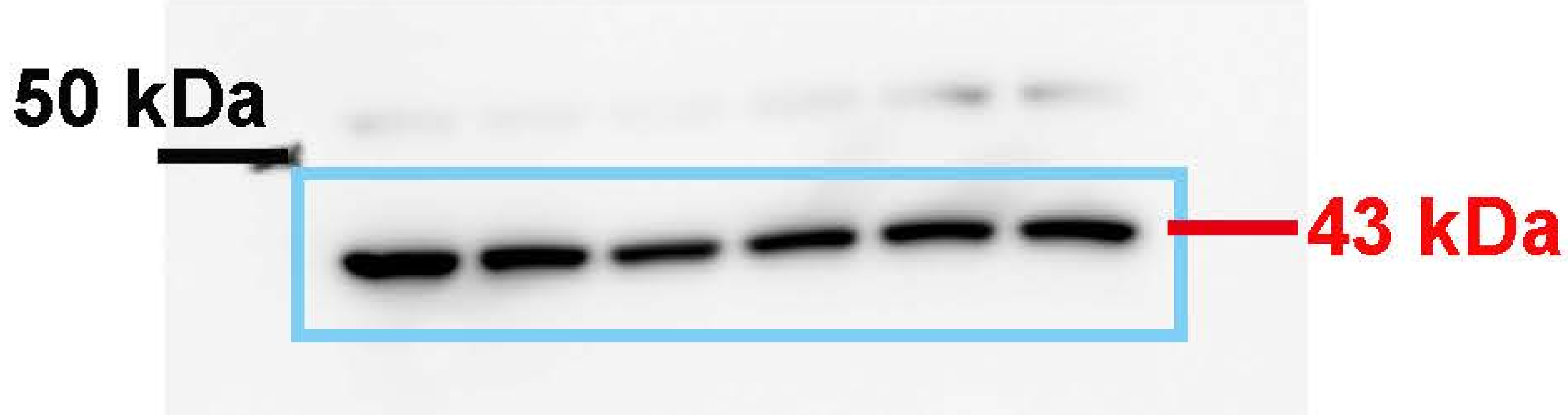

Fig 5A. β-actin

The images were scanned with ImageQuantTM LAS 4000 mini.

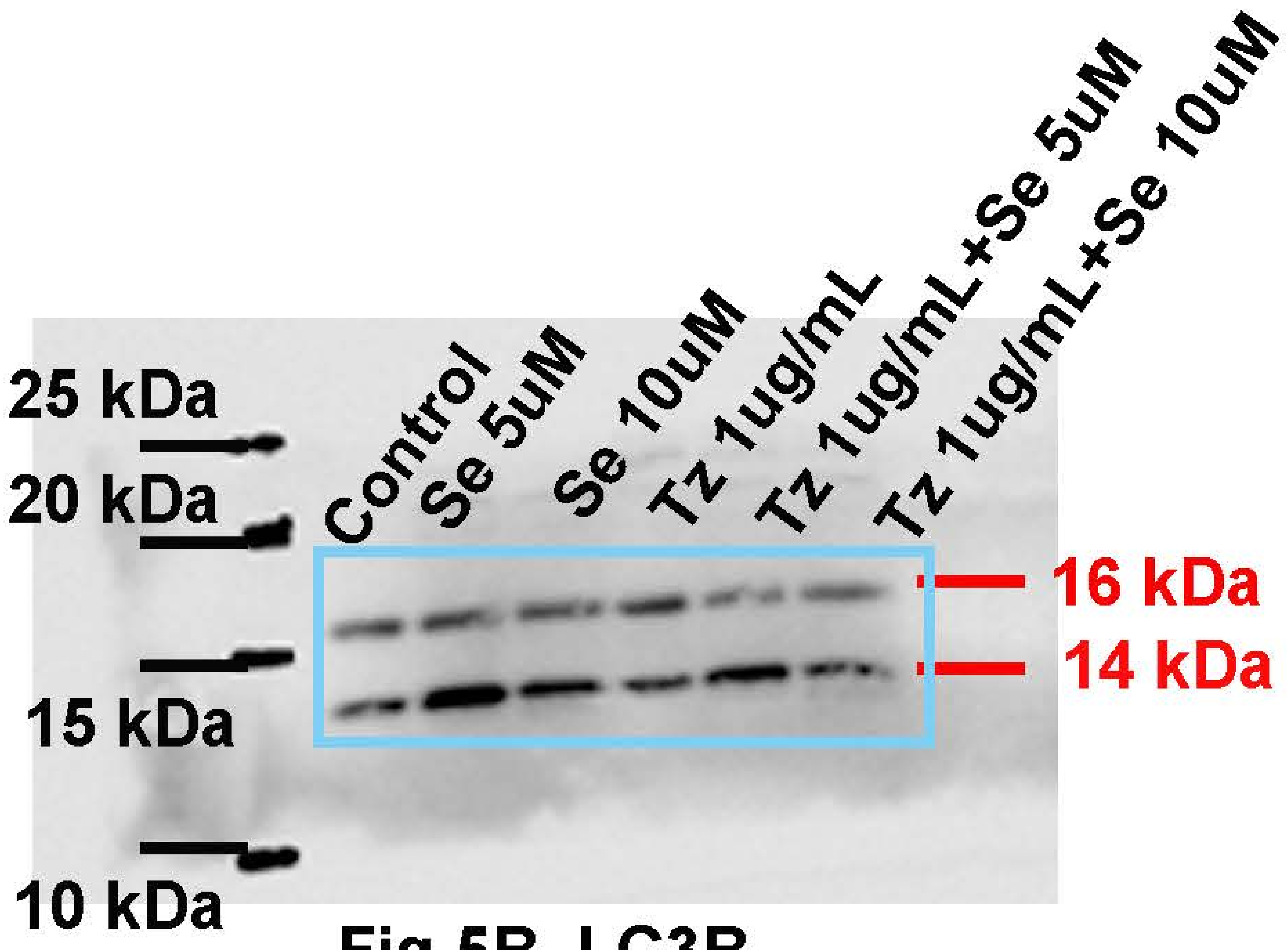

Fig 5B. LC3B

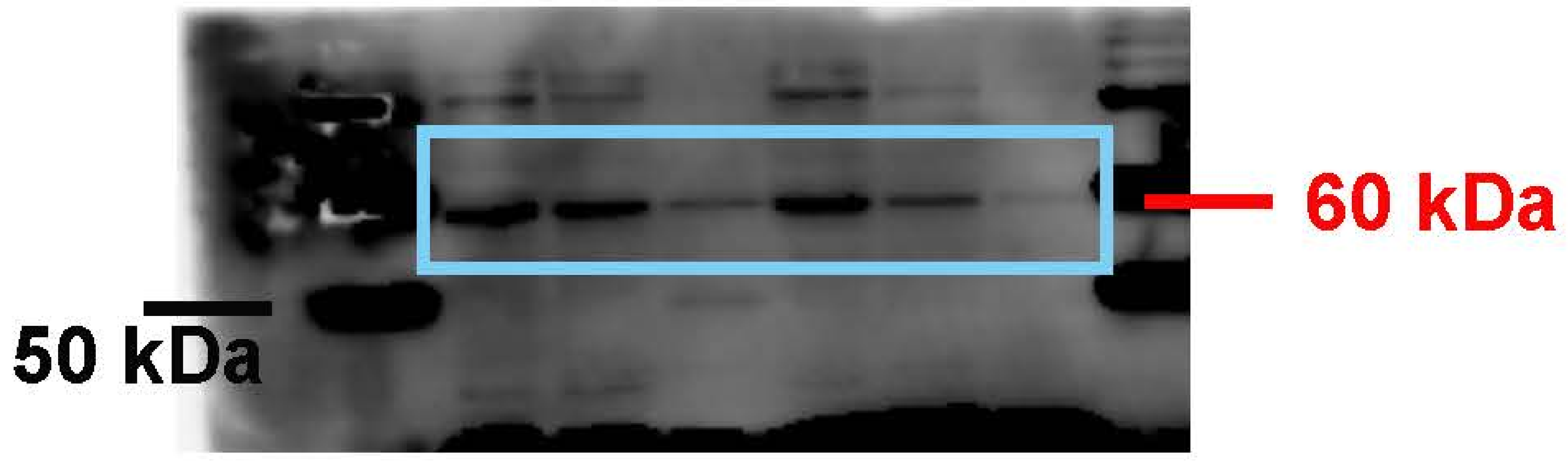

Fig 5B. Beclin-1

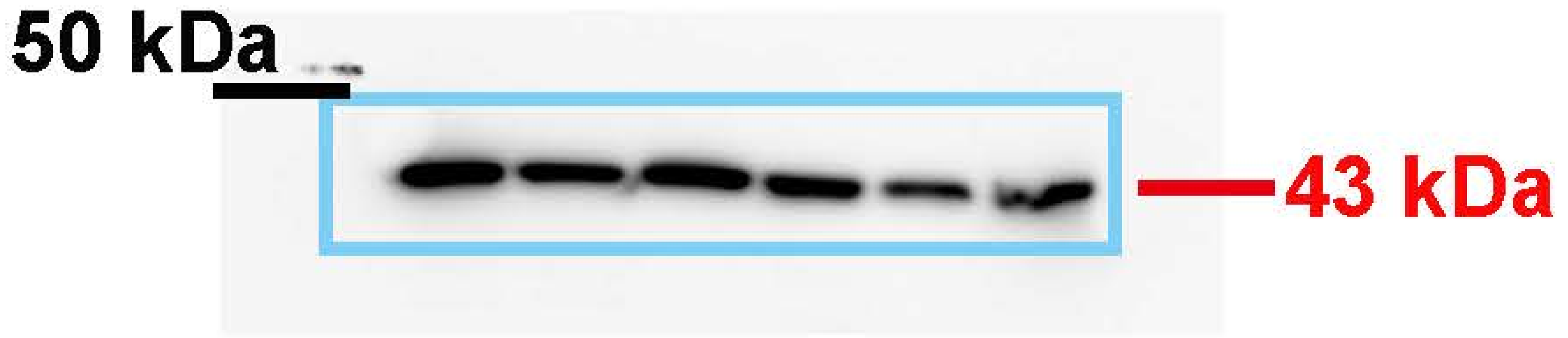

Fig 5B. β-actin

The images were scanned with ImageQuantTM LAS 4000 mini.
